# Supplementary material for: Structural insight into Pichia pastoris fatty acid synthase
Source: Sci Rep. 2021 May 7;11:9773. doi: 10.1038/s41598-021-89196-2 (PMC8105331; doi:10.1038/s41598-021-89196-2)
Supplement: Supplementary file 1 — Supplementary Information. [file 41598_2021_89196_MOESM1_ESM.pdf]

Structural insight into *Pichia pastoris* fatty acid synthase

Joseph S Snowden, Jehad Alzahrani, Lee Sherry, Martin Stacey, David J. Rowlands, Neil A Ranson and Nicola J Stonehouse

Supplementary information

Supplementary Table S1 (related to Figure 1). Quantitative parameters related to cryo-EM data collection.

	**FAS**
Microscope	FEI Titan Krios
Camera	Falcon III
Voltage (kV)	300
Pixel size (Å)	1.065
Exposure rate (e^-/Å^2/s)	46.2
Total dose (e^-/Å^2)	60.1
Number of frames	40
Defocus range (µm)	–0.8 to –3.0
Number of micrographs	3643
Acquisition software	Thermo Scientific EPU
Motion correction	MotionCor2
CTF estimation	GCTF
Image processing	Relion 3.0
Particles picked (including non-FAS particles)	551,674
Particles picked (excluding non-FAS particles)	163,198
Particles contributed	37,054
B-factor	-112
Resolution (FSC=0.143) (Å)	3.1
Map resolution range (Å)	2.7 – 4.9

Supplementary Table S2 (related to Figures 2 and 3). Quantitative parameters and validation statistics related to atomic model building.

	FAS	FAS ACP domain
**PDB ID**	7BC4	7BC5
**Residues modelled**	α: 1-95, 323-534, 599-1751;<br>β: 10 - 2063	α: 139 - 299
**RMSD**		
Bond lengths (Å)	0.0082	0.0055
Bond angles (°)	1.20	1.32
**Validation**		
All-atom clashscore	5.38	7.83
Molprobity score	1.77	1.92
EMRinger score	3.25	0.92
Rotamer outliers (%)	1.19	0.76
**Ramachandran plot**		
Favoured (%)	93.61	91.82
Allowed (%)	6.39	8.18
Outliers (%)	0.0	0.0

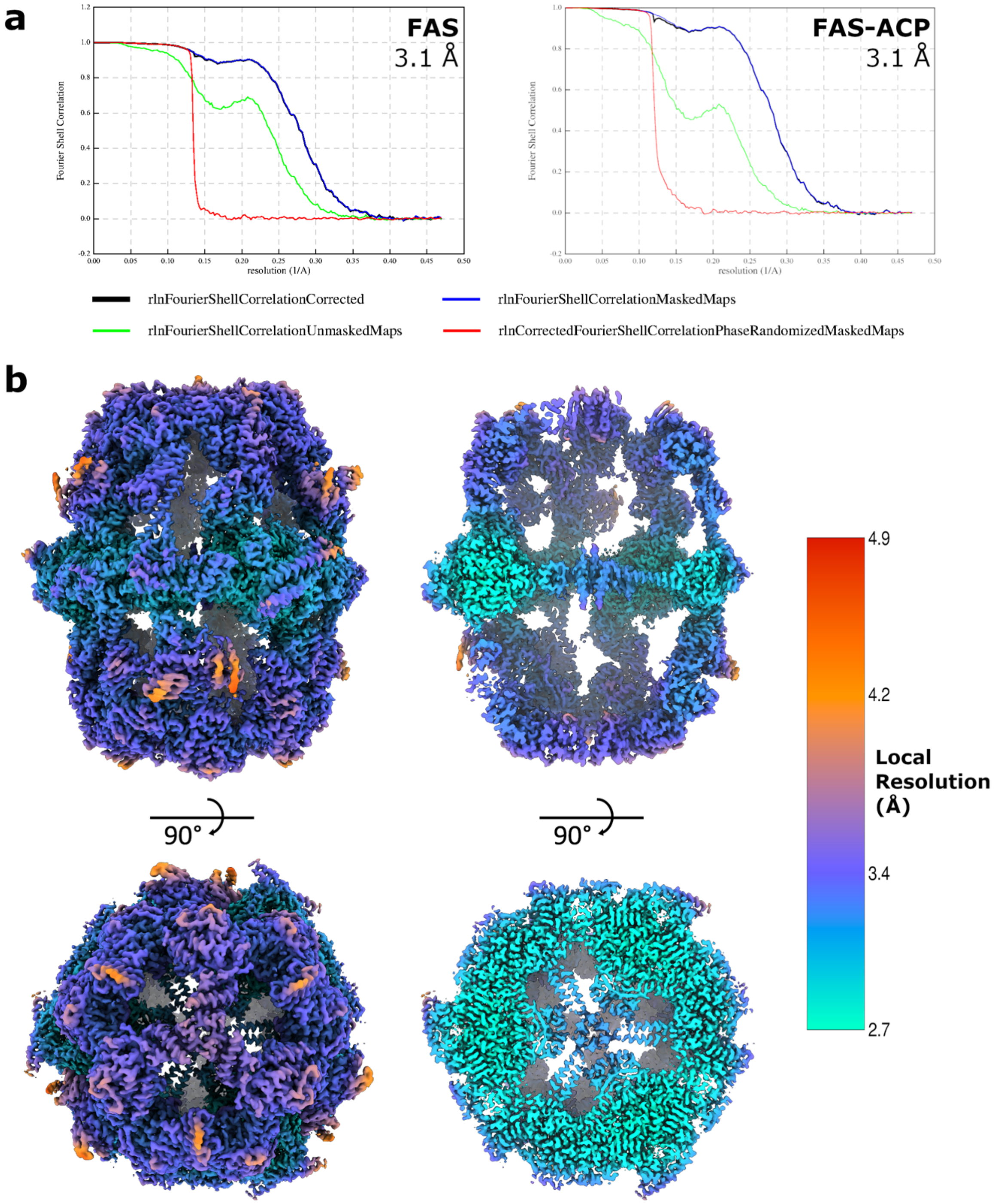

Supplementary Figure S1 (related to Figures 1 and 2). The 3.1-Å resolution reconstruction of *P. pastoris* FAS. **(A)** Fourier shell correlation (FSC) plots for the full symmetric FAS reconstruction (FAS, left) and the reconstruction of FAS from the focussed class containing improved ACP density (FAS-ACP, right). The resolution for each map was determined using the FSC = 0.143 criterion. **(B)** Isosurface

representation of the FAS reconstruction, filtered by local resolution and coloured according to the local resolution scale indicated.

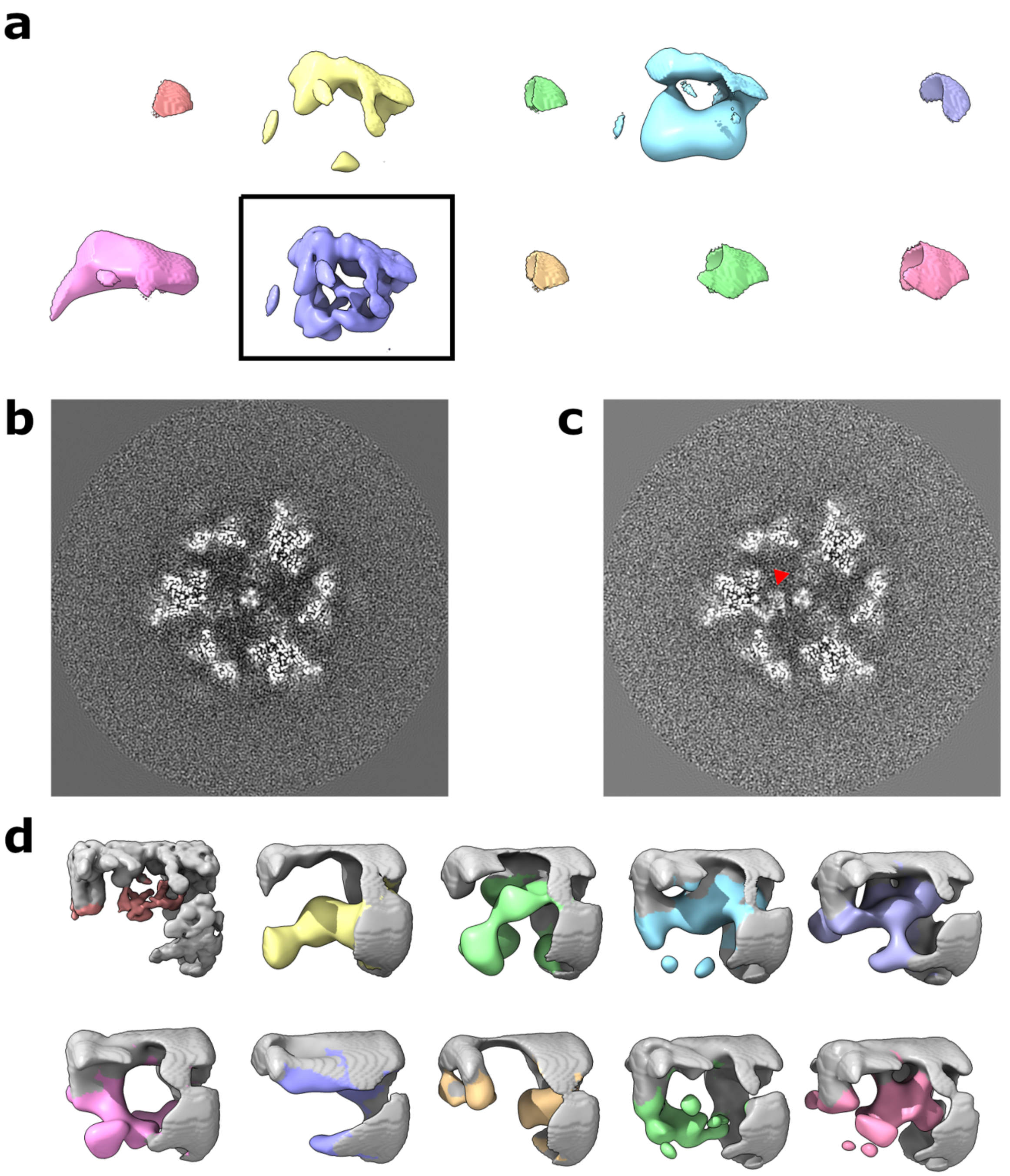

Supplementary Figure S2 (related to Figure 2). (A) All classes from focussed 3D classification of the ACP-containing region of the reaction chamber. All classes are shown at the same contour level. Particles from the boxed class were used for asymmetric reconstruction of FAS. **(B)** Slice through the sharpened D3-symmetric density map (box size 460 pixels) of FAS at the level of the KS and ACP domains. **(C)** Slice through the sharpened asymmetric reconstruction of FAS (box size 460 pixels) following focussed classification of ACP domain density, at the level of the KS and

ACP domains. The red arrowhead indicates the position of the improved ACP domain density. **(D)** All classes from focussed 3D classification of the ACP-containing region of the reaction chamber using an expanded masked region. Grey regions correspond to density within 5.5 Å of FAS atomic coordinates without the ACP domain (i.e., the outer wall and central platform that surround the ACP domain-containing interior chamber). All classes are shown at the same contour level. Several classes are also shown in Fig 2F.

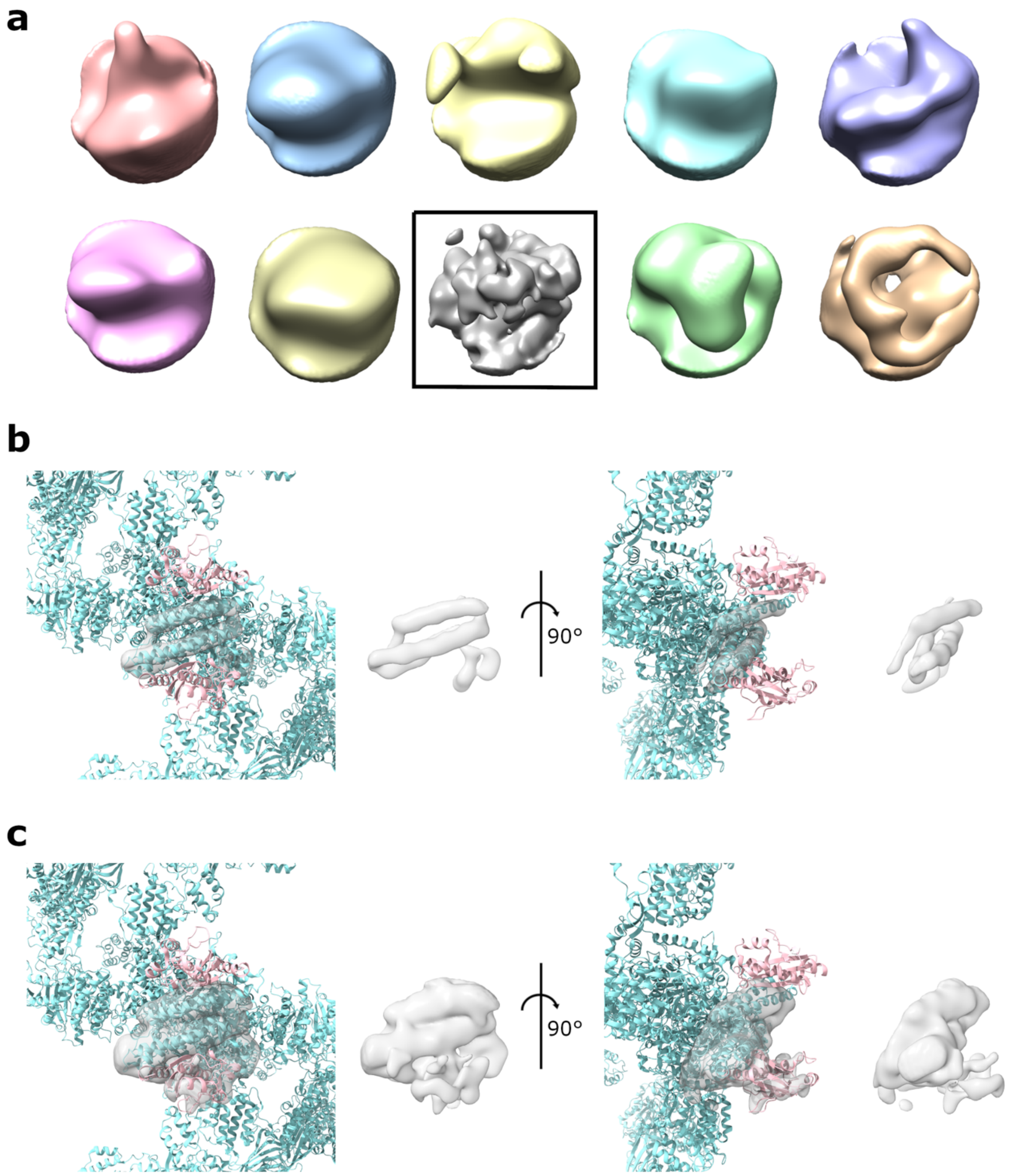

Supplementary Figure S3 (related to Figure 2). Focussed classification of the PPT domain. **(A)** All classes from focussed 3D classification of the region expected to contain PPT domain density. **(B, C)** PPT density-containing class (indicated by the box in (A)) shown at a high contour threshold (only strong density shown) **(B)** and a low contour threshold (weak density also visible) **(C)**. Density is shown from two different viewing angles, either alone or overlaid with the model of *S. cerevisiae* FAS (PDB 6TA1)[1] which contains atomic coordinates for the PPT domain (highlighted in pink).

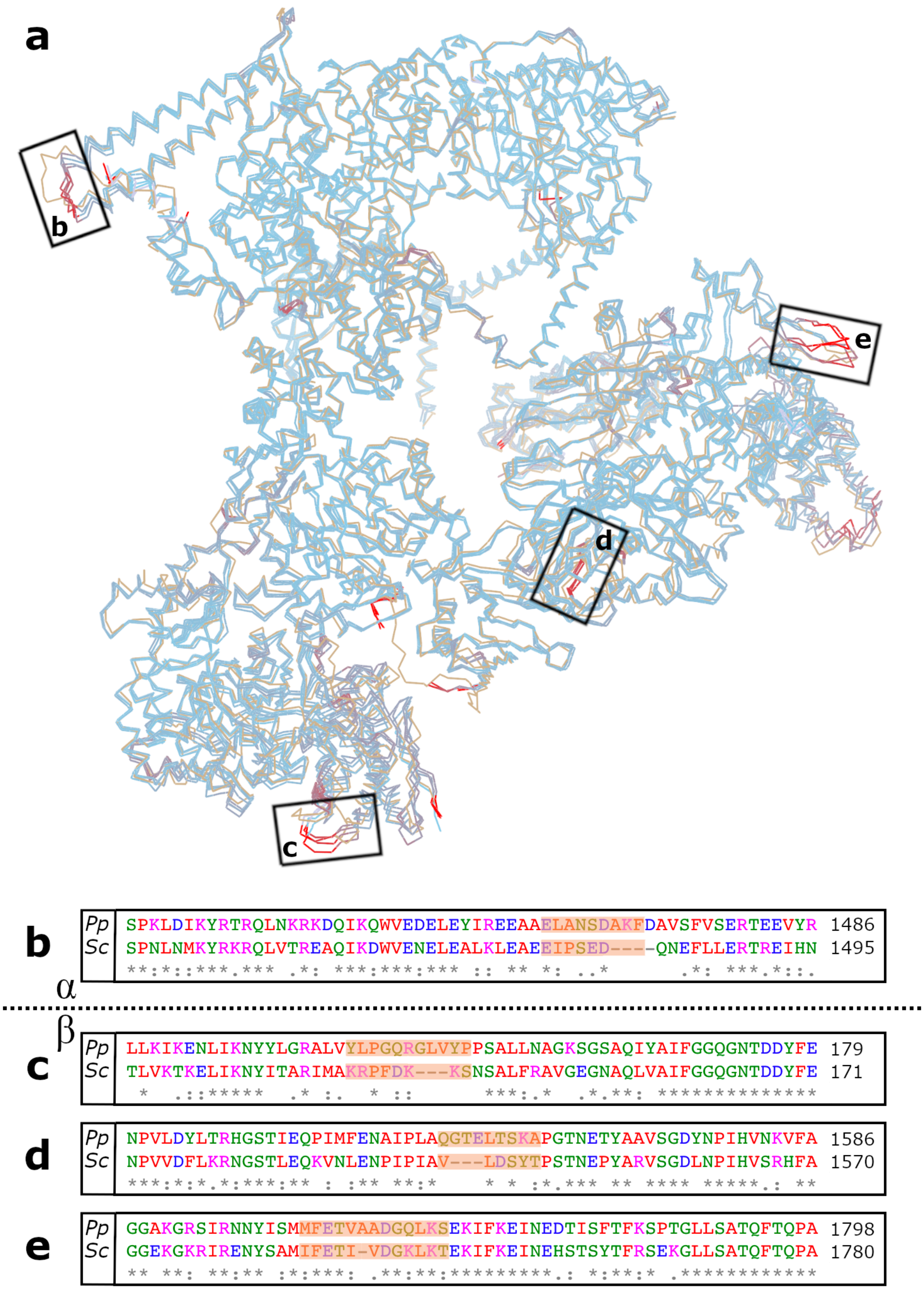

Supplementary Figure S4 (related to Figure 3). Sections of primary sequence alignments between *P. pastoris* FAS and *S. cerevisiae* FAS, corresponding to regions of high structural variation. **(A)** The atomic model of *P. pastoris* FAS (orange) is shown

overlaid with various *S. cerevisiae* FAS models (PDB 6TA1[1], 6QL6[2], 6QL9[2], 2UV8[3]; blue, red segments indicate high Cα RMSD [≥15 Å] compared to the *P. pastoris* FAS model). Segments of the backbone that were obviously misaligned (e.g. due to missing residues) were removed for clarity. Labels on the structural overlay correspond to the primary sequence alignments of α subunits **(B)** and β subunits **(C-E)**. Sequence alignments between *P. pastoris* FAS (UniProt references C4QY10-1 [α subunit], C4QVT8-1 [β subunit]) and *S. cerevisiae* FAS (UniProt references P19097-1 [α subunit], P07149-1 [β subunit]) were performed using Clustal Omega[4] with default parameters, coloured according to the default Clustal colouring scheme. Sections of primary sequence corresponding to the areas of high structural variability in **(A)** are highlighted in red. *Pp* – *P. pastoris* FAS, *Sc* – *S. cerevisiae* FAS.

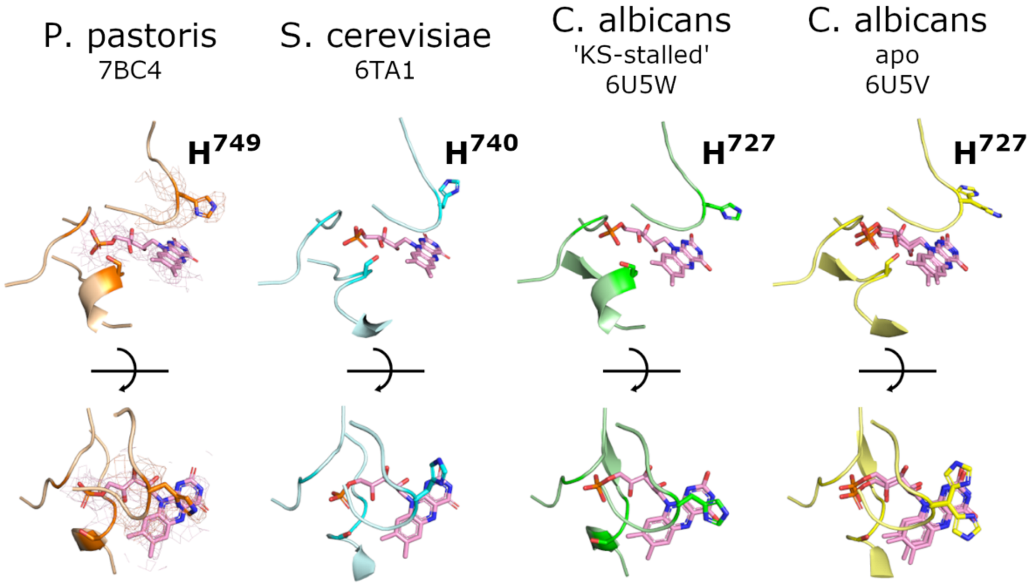

Supplementary Figure S5 (related to Figure 4). Comparison of ER domain catalytic histidine between FAS from different yeast strains[1,5]. Structures for both KS (ketoacyl synthase)-stalled and apo states of *C. albicans* FAS are shown.

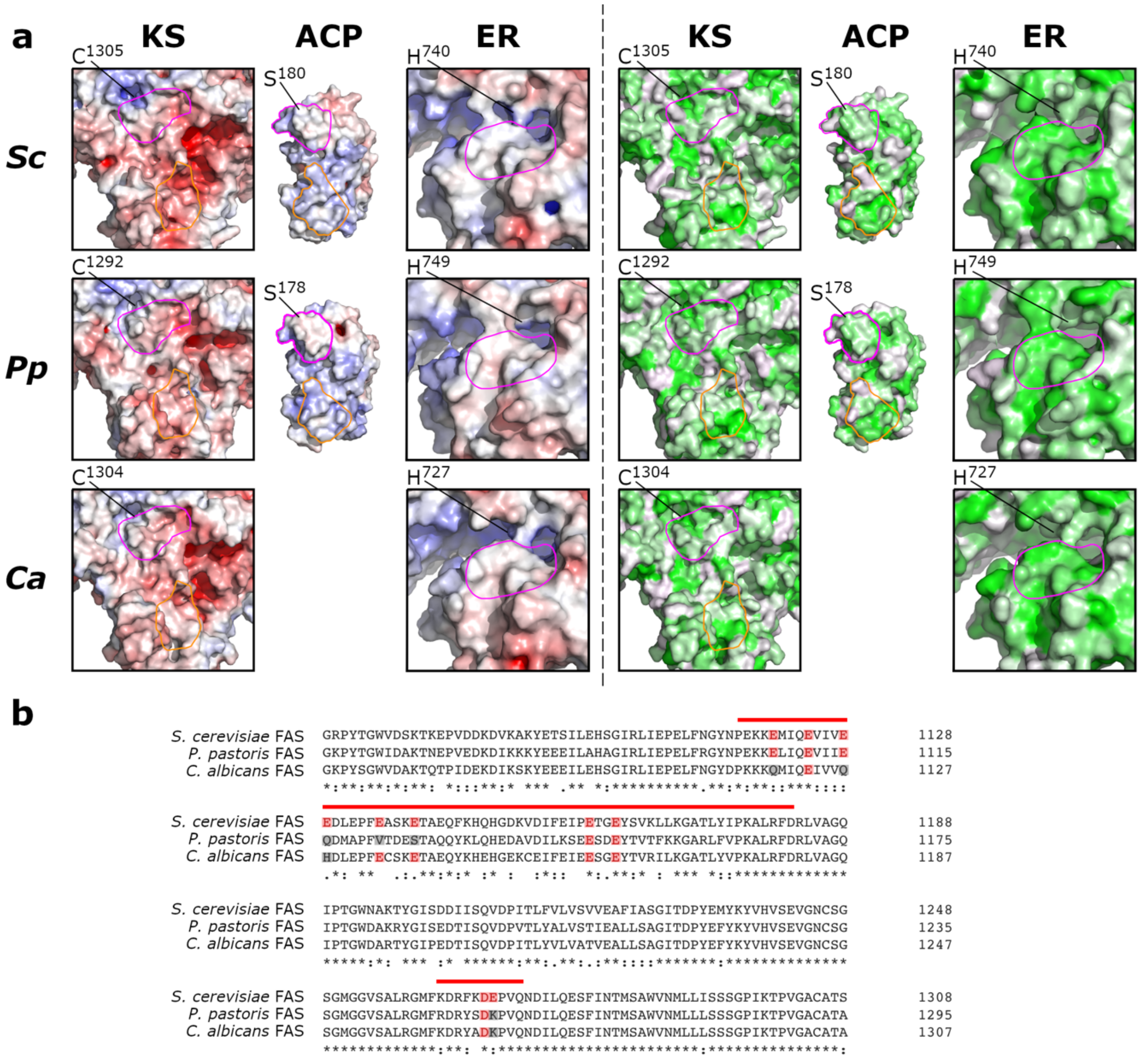

Supplementary Figure S6 (related to Figure 2). Analysis of ACP-KS and ACP-ER interactions. **(A)** Molecular surfaces of ketoacyl synthase (KS), acyl carrier protein (ACP) and enoyl reductase (ER) domains from *S. cerevisiae* (Sc; PDB: 6TA1)[1], *P. pastoris* (Pp) and *C. albicans* (Ca; PDB 6U5V)[5] FAS complexes. Surfaces are coloured by surface charge using PDB2PQR[6] and APBS[7] (left; blue – positive charge, red – negative charge) or hydrophobicity according to the Eisenberg hydrophobicity scale[8] (right; green – hydrophobic). Approximate interfaces are outlined in magenta and orange. ACP domain side chains were not resolved in the *C. albicans* FAS model, and therefore not included in the structural comparison. **(B)** A sequence alignment between FAS α subunits from *S. cerevisiae* (UniProt reference P19097-1), *P. pastoris* (UniProt reference C4QY10-1), and *C. albicans* (UniProt reference P43098-1) was performed using Clustal Omega[4] with default parameters. Parts of the primary sequence of the KS domain that contribute to the ACP-KS interface are indicated with

the red horizontal line. Key acidic residues (as previously identified[5]) are highlighted in red, with altered residues highlighted in grey for *P. pastoris* FAS and *C. albicans* FAS.

References

1. Joppe, M. *et al.* The resolution revolution in cryoEM requires high-quality sample preparation: a rapid pipeline to a high-resolution map of yeast fatty acid synthase. *IUCrJ* **7**, 220–227 (2020).
2. Singh, K. *et al.* Discovery of a regulatory subunit of the yeast fatty acid synthase. *Cell* **180**, 1130–1143.e20 (2020).
3. Leibundgut, M., Jenni, S., Frick, C. & Ban, N. Structural basis for substrate delivery by acyl carrier protein in the yeast fatty acid synthase. *Science* **316**, 288–290 (2007).
4. Sievers, F. *et al.* Fast, scalable generation of high-quality protein multiple sequence alignments using Clustal Omega. *Mol. Syst. Biol.* **7**, 539 (2011).
5. Lou, J. W., Iyer, K. R., Hasan, S. M. N., Cowen, L. E. & Mazhab-Jafari, M. T. Electron cryomicroscopy observation of acyl carrier protein translocation in type I fungal fatty acid synthase. *Sci. Rep.* **9**, 12987 (2019).
6. Dolinsky, T. J. *et al.* PDB2PQR: expanding and upgrading automated preparation of biomolecular structures for molecular simulations. *Nucleic Acids Res.* **35**, W522–W525 (2007).
7. Baker, N. A., Sept, D., Joseph, S., Holst, M. J. & McCammon, J. A. Electrostatics of nanosystems: application to microtubules and the ribosome. *Proc. Natl. Acad. Sci. USA* **98**, 10037–10041 (2001).
8. Eisenberg, D., Schwarz, E., Komaromy, M. & Wall, R. Analysis of membrane and surface protein sequences with the hydrophobic moment plot. *J. Mol. Biol.* **179**, 125–142 (1984).
